# Social Media Use Profiles in Adolescents: A Cluster Analysis of Interpersonal Difficulties and Social Anxiety

**DOI:** 10.3390/bs16040619

**Published:** 2026-04-21

**Authors:** Natalia Morán-Pallero, Elena Felipe-Castaño, Margarita Gozalo, Marina Rojas-Valverde

**Affiliations:** 1 Consejería de Educación, Junta de Extremadura, 10002 Caceres, Spain; nmoranp01@educarex.es; 2 Department of Psychology and Anthropology, Faculty of Teacher Training College, University of Extremadura, 10071 Caceres, Spain; mrojasva@alumnos.unex.es; 3 Department of Psychology and Anthropology, Faculty of Sport Science (Psychology Laboratory), University of Extremadura, 10005 Caceres, Spain; mgozalo@unex.es

**Keywords:** social media, interpersonal difficulties, social anxiety, adolescence, cluster analysis

## Abstract

Social media (SM) are virtual platforms for interpersonal interactions and play a significant role in the well-being of adolescents. It is therefore essential to understand how they use SM and how this use relates to interpersonal difficulties and social anxiety. The primary aim of this study was to examine SM use profiles together with interpersonal difficulties and social anxiety in a sample of adolescents. The sample comprised 304 adolescents aged 16–18 years, selected through probabilistic sampling: 170 (55.9%) were female and 134 (44.1%) were male. Four SM use profiles were identified, according to sex, time spent on SM, and number of accounts. Differences in interpersonal difficulties and social anxiety were observed across profiles, particularly among adolescents who showed intensive SM-centred use of the internet. Differences between males and females were also evident. The findings and their implications for the development of programmes aimed at encouraging healthy SM use among adolescents with a special focus on interpersonal well-being are discussed.

## 1. Introduction

Social media (SM) are defined as virtual communities that enable users to meet new people, interact with friends, share information, and comment on multimedia content (Hong et al., 2020; Shahnawaz & Rehman, 2020). Their main appeal lies in the high degree of interactivity they offer, which allows the internet user to become an active agent in the communication process (Chóliz, 2017). In this context, SM use has become an established part of everyday life among adolescents and young people. According to data from the Spanish National Statistics Institute (INE, 2021), the frequency and duration of SM use is highest among the 15–24 age group (Feliciano-García et al., 2019; O’Day & Heimberg, 2021).

SM fulfil predominantly recreational and relational functions by allowing users to build and maintain interpersonal relationships (Ballesta Pagán et al., 2021; Martínez & Sánchez, 2016). They have expanded the traditional physical socialisation environments to include the virtual realm. As a result, the lack of an active SM presence, particularly during adolescence, can lead to exclusion from an important part of peer-group social life (Metzler & Scheithauer, 2015).

Adolescence is a developmental stage during which interpersonal relationships among peers constitute a fundamental form of social capital and provide an important source of emotional support. However, this period is also characterised by heightened emotional vulnerability, marked by social inhibition, concerns about being judged negatively and self-image insecurities, all of which contribute to increased levels of social anxiety (Beesdo-Baum et al., 2012).

Against this backdrop, the question arises as to how these psychosocial dynamics unfold in the virtual environment. SM may function as potential sources of social support, attention and social interaction processes (Dumas et al., 2020; Košir et al., 2016). From this perspective, several studies indicate that different patterns of SM use, characterised by varying levels of interaction and participation, are associated with higher levels of psychological well-being and perceived social support (Godard & Holtzman, 2024). Adolescents who perceive themselves as capable of establishing and maintaining meaningful relationships online tend to report broader and more diverse friendship networks, online as well as offline (Subrahmanyam et al., 2015).

However, recent evidence underscores that the impact of digital technology use may vary depending on individual characteristics and underlying psychological processes (Fabio & Suriano, 2024). In particular, social anxiety and interpersonal difficulties may influence how adolescents use social media: those with higher social anxiety may avoid face-to-face interactions, while those with interpersonal difficulties may experience deficits in social skills. Social media can thus serve both as a compensatory environment and an avoidance strategy, contributing to distinct patterns of use in terms of intensity and general engagement with SM. In this regard, the meta-analysis by Godard and Holtzman (2024) found that different patterns use of SM is associated with variations in mental health indicators, including levels of anxiety and well-being.

Intensive SM use may also interfere with activities that are fundamental to the well-being of adolescents, such as engaging in face-to-face social interaction and physical activity, and getting adequate rest (Malo-Cerrato et al., 2018; Morán-Pallero & Felipe-Castaño, 2021). Recent reviews confirm that greater amounts of screen time and higher engagement with SM are associated with sleep disturbance and increased symptoms of anxiety and depression (Santos et al., 2023; Yu et al., 2024).

The body of research on the relationship between SM use and mental health has yielded mixed findings. Numerous studies report correlations between greater time spent on SM and reduced psychological well-being, alongside increased symptoms of anxiety and depression (O’Day & Heimberg, 2021; Seabrook et al., 2016; Woods & Scott, 2016). In addition, recent systematic reviews indicate that problematic SM use constitutes a risk factor for anxiety—including social anxiety—and interpersonal difficulties (Du et al., 2024; Brand et al., 2024).

However, other studies suggest that time spent on SM is not the sole problematic factor and that additional variables need to be taken into consideration, such as the number of SM platforms used, the number of active accounts, the simultaneous use of multiple platforms, and perceived ‘online social support’ (Boer et al., 2021; Coyne et al., 2020; Luchtefeld & Jordan, 2022; Primack et al., 2017; Vannucci & McCauley Ohannessian, 2019). Along similar lines, longitudinal reviews indicate that certain usage profiles characterised by greater involvement in SM use, engagement across multiple platforms, and pre-existing psychosocial adjustment difficulties are associated with a greater risk of developing problematic use and internalising symptoms (Pazdur et al., 2025). This impact appears to be particularly pronounced among females (Avci et al., 2024; Diggins et al., 2024; Niskier et al., 2024; Urbán et al., 2024) and among adolescents with prior interpersonal difficulties, who may use SM to avoid or to compensate for the lack of face-to-face interaction (Lee & Chiou, 2013; Malo-Cerrato et al., 2018; She et al., 2023).

In line with the previous literature, the variables selected for the clustering procedure were chosen to capture both the quantitative and qualitative dimensions of adolescents’ engagement with social media. Specifically, time-related indicators (i.e., daily time spent on social media and its proportion relative to total internet use) have consistently been identified as key markers of intensity of use and potential risk for problematic outcomes (Santos et al., 2023; Yu et al., 2024). In addition, the number of social media accounts reflects the multiplicity of platforms used, which has been associated with higher levels of psychological distress and greater involvement in SM use (Boer et al., 2021; Primack et al., 2017; Vannucci & McCauley Ohannessian, 2019). Finally, sex was included as a grouping variable given the well-documented gender differences in patterns of social media use and their psychological correlates, with female adolescents showing higher vulnerability to internalising symptoms and problematic SM use (Avci et al., 2024; Diggins et al., 2024; Niskier et al., 2024).

Despite the growing body of research on SM use in adolescence, several important gaps remain. First, many studies focus on isolated variables, such as the amount of time spent online or the presence of psychological symptoms, instead of adopting an integrative approach that captures the co-occurrence of sociodemographic characteristics, patterns of connection and SM-related behaviours within the same analytical framework. Second, although the relationship between SM use and anxiety has been widely documented, comparatively less attention has been paid to social anxiety and interpersonal difficulties, which are key dimensions during adolescence. Furthermore, while person-centred approaches have gained increasing attention, relatively fewer studies have examined distinct SM use profiles using multivariate techniques such as cluster analysis that simultaneously incorporate both behavioural and structural variables.

In this context, the primary aim of the present study was to identify SM use profiles among adolescents and to examine differences between these profiles in interpersonal difficulties and social anxiety. The following hypotheses were proposed: (a) distinct SM use profiles would be identified among adolescents based on sex, time spent on SM relative to overall internet use, daily time spent on their preferred SM platform, and number of accounts on the preferred platform, and (b) differences between profiles would emerge in interpersonal difficulties and social anxiety, such that profiles characterised by intensive and SM-centred use would show higher levels of interpersonal difficulties and social anxiety.

## 2. Materials and Methods

### 2.1. Participants

The sample comprised 304 adolescents aged 16–18 years (*M* = 17.16, *SD* = 0.81); of whom, 55.9% (*n* = 170) were female and 44.1% (*n* = 134) were male. This age range is consistent with the developmental stage of late adolescence, even when participants are enrolled in the first year of university. Participants were selected using a multistage stratified cluster sampling procedure, with age (16–18 years) as the stratification variable and educational institution as the clustering variable, based on the selection of naturally occurring class groups. Eight secondary schools and four university faculties in Spain were selected. Within each institution, two class groups were randomly selected. Participants met the inclusion criteria of being aged 16–18 years and providing informed consent. Regarding educational level, 37.5% (*n* = 114) were in the first year of the final two-year cycle of the Spanish secondary education system, 22% (*n* = 67) were in its final year, and 40.5% (*n* = 123) were in the first year of university. A 5% margin of error was assumed in determining the sample size and selection procedure.

### 2.2. Instruments

Sociodemographic data: Information was collected on age, sex, educational level, preferred SM platform, time spent on the preferred platform relative to total internet use, daily time spent (in hours) and number of accounts on the preferred platform.

Inventory of Interpersonal Problems (IIP-32, Barkham et al., 1996; adapted from IIP-64 by Horowitz et al., 1988): The Spanish 32-item adaptation by Salazar et al. (2010) was used. This inventory assesses individuals’ difficulties in their relationships with others through eight scales grouped into four dimensions, each comprising two opposing poles. It is structured as a list of problems, and respondents are asked to indicate whether they have experienced specific difficulties when interacting with a significant person in their life over the past two weeks. Responses are recorded on a five-point Likert-type scale ranging from 0 (not at all) to 4 (extremely). The 32 items are distributed across eight interpersonal problem scales (internal consistency coefficients obtained in the present study are reported): Domineering/Controlling (*α* = 0.674), Vindictive/Self-Centred (*α* = 0.799), Cold/Distant (*α* = 0.794), Non-assertive (*α* = 0.828), Overly Accommodating (*α* = 0.738), Socially Inhibited (*α* = 0.628), Self-Sacrificing (*α* = 0.736), and Intrusive/Needy (*α* = 0.668). The inventory also provides a total score for interpersonal difficulties (*α* = 0.848). Higher scores on a given scale indicate greater interpersonal difficulties in interactions with others. Table 1 presents an interpretation of each IIP scale.

Social Anxiety Questionnaire for Adults (SAQ-A30, Caballo et al., 2010): The instrument assesses social anxiety as a dimensional construct. It comprises 30 items rated on a five-point Likert-type scale ranging from 1 (no or very little discomfort, tension, or nervousness) to 5 (very high or extreme discomfort, tension, or nervousness). The questionnaire includes five social anxiety factors: public speaking/interaction with authority figures (*α* = 0.872); interaction with strangers (*α* = 0.831); interaction with people I find attractive (*α* = 0.832); assertive expression of annoyance, displeasure, or anger (*α* = 0.771); and fear of embarrassment or appearing foolish (*α* = 0.697). The original items that used the term the “opposite sex” (Items 4 and 23) were modified to refer instead to “people I find attractive” to avoid discriminating against non-heterosexual individuals, as the present study did not aim to assess sexual orientation.

### 2.3. Procedure

The study was approved by the Bioethics Committee of the University of Extremadura (Reference No. 169/2021). The research was conducted in accordance with the ethical principles of the 1975 Declaration of Helsinki and its 2008 revision. It also complied with Spanish Organic Law 3/2018 of 5 December on the Protection of Personal Data and Guarantee of Digital Rights.

In line with these requirements, all data were treated confidentially, access was restricted to the research team, and the data were used solely for the purposes of the present study.

For participants under the age of 18 (16–17 years), the management teams of the selected educational institutions were first contacted to obtain institutional authorisation. Subsequently, a parental consent form was sent to the parents or legal guardians of the minors. On the day of the data collection, students provided their informed consent in the classroom. For participants aged 18 years or older, teaching staff were contacted, and informed consent was obtained directly from participants.

For all participants, the questionnaire battery was administered in groups by class during a single assessment session lasting approximately 45 min. Instructions for completing the questionnaires were provided, and participants were reminded their responses were anonymous and confidential, that the data would be used exclusively for research purposes, and that participation was voluntary.

### 2.4. Data Analysis

Data coding and analysis were conducted in IBM SPSS Statistics 25 for Windows (SPSS Inc., Chicago, IL, USA). The analyses included tests of normality using the Kolmogorov–Smirnov (K–S) test, internal consistency analyses using Cronbach’s *α*, and descriptive statistics. The variables of interest approximated a normal distribution; therefore, parametric tests were used.

An exploratory two-step cluster analysis was conducted as it allows the inclusion of both categorical and continuous variables. The selection of clustering variables was theoretically driven and included both behavioural indicators of social media use (time of use, number of profiles, and relative use compared to other internet activities) and sex, which was incorporated as a structural variable given its well-documented association with differential patterns of social media use during adolescence. This approach aimed to identify naturally occurring profiles that reflect the combined influence of usage patterns and sociodemographic characteristics.

The final solution was subsequently externally validated by comparing the resulting clusters on interpersonal difficulties (IIP-32) and social anxiety (SAQ-A30), which were not included in the clustering procedure. The two-step approach is suitable for datasets containing mixed variable types (categorical and continuous) and enables the number of clusters to be automatically selected using information criteria and distance measures (Chiu et al., 2001).

The algorithm used a log-likelihood distance measure and Schwarz’s Bayesian criterion (BIC) to determine the optimal cluster solution. Model quality was assessed using the silhouette coefficient (cluster separation). Based on the final solution, the resulting profiles were characterised using category distributions for each clustering variable. Subsequently, as the assumption of homogeneity of variances was not met for some scales, comparative analyses using Welch’s ANOVA and Games–Howell robust post hoc tests were conducted to examine differences between clusters in interpersonal difficulties and social anxiety. Effect size was estimated using *ω*^2^ (omega squared), calculated from the classical ANOVA.

Across all hypothesis tests, a 95% confidence interval was applied, and a significance level of *p* < 0.05 was adopted.

## 3. Results

All participants reported using SM and maintaining active accounts. The most frequently used and preferred platform was Instagram (86.5%; *n* = 263), followed at a considerable distance by TikTok (8.9%; *n* = 27) and X (4%; *n* = 12). No sex-related differences were found in the distribution of preferred platform (*χ*^2^ = 3.326; *df* = 3; *p* = 0.344). Regarding the number of accounts on the most frequently used platform, having a single account was most common (62.4%, *n* = 189), 29.4% (*n* = 89) had two accounts, and only 25 participants (8.4%) had three or more accounts. No sex-related differences were found in the number of accounts (*χ*^2^ = 4.910; *df* = 5; *p* < 0.427).

The two-step cluster analysis supported a four-cluster solution as the best fit. BIC values decreased up to the four-cluster solution (BIC[4] = 315.238), and the ratio of distance measures for the transition from three to four clusters was high (=2.28), indicating good separation between the three- and four-cluster solutions. The overall silhouette measure was in the mid-range (0.4), suggesting moderate cluster quality (Collins & Lanza, 2010). This result is considered common in social science data involving mixed variable types (quantitative and categorical) and can still support meaningful group-level interpretation (Rousseeuw, 1987). In addition, a small number of cases characterised by extreme values (an outlier group) was retained as unassigned. Table 2 presents descriptive statistics for the clustering variables by cluster.

It should be noted that the identified clusters reflect multivariate configurations of characteristics, and therefore differences between groups may not always be pronounced on individual variables when considered separately.

Regarding between-group differences in interpersonal difficulties (see Table 3), statistically significant differences were found on the IIP-2 (Vindictive/Self-Centred), IIP-6 (Overly Accommodating), IIP-7 (Self-Sacrificing), and IIP-8 (Intrusive/Needy) scales, with small-to-moderate effect sizes (*ω*^2^ ranging from 0.027 to 0.055). The largest effect size was observed for IIP-7 (*ω*^2^ = 0.055), indicating that cluster membership accounted for approximately 5.5% of the variance in this interpersonal dimension. Although this effect size may be considered small to moderate, it may still be meaningful in the context of complex phenomena and should not be dismissed (Lakens, 2013).

Statistically significant between-group differences were found across all SAQ-A30 factors (*p* < 0.05) except Factor 2 (interaction with strangers), with small-to-moderate effect sizes. The largest effect was observed for the public speaking/interaction with authority figures factor (*ω*^2^ = 0.067), followed by fear of embarrassment or appearing foolish (*ω*^2^ = 0.040) (see Table 4). These findings indicate that membership in different SM use profiles accounted for a significant, albeit moderate, proportion of the variance in specific dimensions of social anxiety.

The groups are described below by integrating the characteristics of each profile with the observed differences in interpersonal difficulties and social anxiety. A summary is presented in Table 5.

Group 1 (*n* = 49, 16.2%): This group comprised females characterised by moderate and balanced SM use (*M* = 2.27, *SD* = 1.43); overall, 65.3% (*n* = 32) had a single active account and distributed their online time relatively evenly between SM and other internet activities. This group showed elevated scores on the Overly Accommodating subscale (IIP-6), which characterises individuals who find it difficult to express anger for fear of offending others, and who describe themselves as trusting and easily misled. In terms of social anxiety, this group obtained the second-highest mean score on public speaking/interaction with authority figures, differing significantly from Groups 3 and 4 (*ω*^2^ = 0.067).

Group 2 (*n* = 111, 36.6%): This group comprised females characterised by intensive, multi-account, and SM-centred use. It reported the highest mean daily use (*M* = 3.77 ± 2.22), spent more than half the total internet time on SM, and 44.1% of participants reported having between two and four accounts. This group showed the profile most consistently associated with indicators of interpersonal difficulties and social anxiety. On the IIP, the largest effect sizes were observed for the Self-Sacrificing (IIP-7, *ω*^2^ = 0.055) and Intrusive/Needy (IIP-8, *ω*^2^ = 0.027) scales. This profile is consistent with an excessively affiliative style, characterised by being overly helpful and giving, generous, affectionate, trusting, and permissive. It also reflects a strong need for connection with others, a tendency towards inappropriate self-disclosure, attention-seeking, and difficulty spending time alone. In addition, this group obtained significantly higher scores on public speaking/interaction with authority figures (*ω*^2^ = 0.067) and fear of embarrassment or appearing foolish (*ω*^2^ = 0.040).

Group 3 (*n* = 79, 26.1%): This group comprised males characterised by intensive use in terms of daily hours (*M* = 3.39 ± 1.90) and by spending more than half of their online time on SM. 72.2% had only one account on their preferred platform, and this group obtained the lowest scores on interpersonal difficulties. Although the interpersonal profile suggested self-sacrificing and affiliative tendencies, scores were significantly lower than those observed in Groups 1 and 2. In terms of social anxiety, this group appeared less sensitive to social evaluation, with significantly lower scores than Group 1 on public speaking (Factor 1) and lower scores than Group 2 on fear of embarrassment or appearing foolish (Factor 5).

Group 4 (*n* = 51, 16.8%): This group comprised males characterised by moderate (*M* = 2.27 ± 1.02) and instrumental SM use. Participants reported either one (68.6%) or two (31.4%) active accounts, and most devoted the same amount of time or less to SM than to other online activities. Between-cluster comparisons indicated a significantly higher score only on the Vindictive/Self-Centred subscale (IIP-2) compared with Group 1, with a small-to-moderate effect size. This dimension describes interpersonal difficulties characterised by mistrust and suspicion towards others, as well as a limited capacity to attend to others’ needs and well-being. With respect to social anxiety, this group reported the lowest scores across all factors except for interaction with strangers which showed the second-lowest mean score.

Outlier group (unassigned cases) (*n* = 13, 4.3%): The analysis also identified a small set of cases that were not assigned to any cluster. This group included females and males and was characterised by extremely high SM use (*M* = 5.38 ± 2.18)—although they also engaged in other online activities—and a significantly higher number of active accounts compared with the other groups (*M* = 3.46 ± 1.85). These participants showed very intensive use in terms of hours spent online, had multiple accounts but did not devote their online time exclusively to SM. Due to the small sample size, these cases were not included in the comparative analyses between groups.

## 4. Discussion

The primary aim of this study was to identify SM use profiles among adolescents and to examine differences between these profiles in interpersonal difficulties and social anxiety.

The most widely used platform was Instagram, a general-purpose SM platform that allows users to create personal profiles and interact with others (Gracia-Granados et al., 2020; Hong et al., 2020). Females reported spending more time on SM and maintaining a greater number of accounts across platforms than males, consistent with previous research (Boursier et al., 2020; Hassan & Afzal, 2022; Morán-Pallero & Felipe-Castaño, 2021).

The two-step cluster analysis identified four distinct SM use profiles in the adolescent sample. These profiles differed in intensity of use, the centrality of SM within overall internet use, and the number of active accounts, and also showed sex-related differences. The profiles were characterised as integrated social use, intensive-relational social use, intensive-instrumental social use, and peripheral social use. These findings suggest that adolescent SM use is not homogeneous but is organised into qualitatively distinct profiles or patterns of engagement. Our findings were consistent with previous research (e.g., Varona et al., 2024), which identified motivation-based user typologies (discreet users, leisure users, socialising users, and hyperconnected users) associated with different levels of risk for problematic use and psychosocial well-being.

The SM use profiles were differentially associated with specific patterns of interpersonal difficulties and social anxiety, with a clear sex-related component. In particular, the group of females with moderate and integrated SM use (Group 1) showed difficulties consistent with a compliant interpersonal style, characterised by inhibition in the expression of anger and fear of interpersonal conflict, as well as difficulties with public speaking and interactions with authority figures. Previous research suggests that this pattern is relatively common among female adolescents and may be related to gender socialisation norms that promote relational harmony and conflict avoidance (Blackstone, 2003).

The group characterised by intensive and SM-centred use comprising females (Group 2), showed the highest levels of interpersonal difficulties relative to the other groups. This profile was marked by a tendency to prioritise others’ needs over one’s own and by more Intrusive/Needy interpersonal tendencies, characterised by inappropriate self-disclosure, attention-seeking, and difficulties related to spending time alone. This pattern may be interpreted as indicating that intensive and SM-centred internet use, together with heightened sensitivity to negative social evaluation, may be associated with avoidance of evaluative in-person situations and a greater reliance on online social interaction. This interpretation is consistent with recent research indicating that female adolescents tend to use SM primarily for relational and emotional purposes, which has been associated with greater exposure to social comparison and interpersonal self-demands (Valkenburg et al., 2022; Varona et al., 2024). From this perspective, intensive relational use may be linked to interpersonal styles marked by fear of rejection, and difficulty setting boundaries, which are consistent with the Self-Sacrificing and Intrusive/Needy profiles described in the IIP.

In contrast, the male-dominated groups who exhibited intensive SM use (Group 3), but low-social-orientation use (Group 4) showed significantly lower levels of interpersonal difficulties across most dimensions, except for the Vindictive/Self-Centred dimension, on which Group 4 scored highest. The Vindictive/Self-Centred subscale describes interpersonal patterns characterised by mistrust, suspicion, and limited concern for others’ needs. In terms of social anxiety, these groups also reported the lowest scores across all factors except interaction with strangers. Psychologically, this pattern may reflect a more instrumental or peripheral use of SM that does not substantially co-occur with interpersonal or social adjustment difficulties. This is consistent with studies suggesting that some adolescent boys may express interpersonal difficulties through more externalising or defensive styles rather than through greater interpersonal involvement (Niu et al., 2023; Zhou et al., 2023).

Regarding social anxiety, the female-dominated groups—both moderate and intensive SM users—reported the highest scores across nearly all dimensions. More specifically, females with intensive SM use showed higher levels of social anxiety in interpersonal interactions, situations involving public scrutiny, and assertive expression of annoyance or anger, as well as greater fear of negative social evaluation, including concerns about feeling embarrassed or appearing foolish.

This pattern is consistent with recent evidence showing that social anxiety is more prevalent among female adolescents, particularly in situations that involve social evaluation, comparison with others, and visibility to peers (Urbán et al., 2024), all of which may be salient in SM contexts. In addition, several longitudinal studies suggest that intensive and highly engaged SM use may be associated with negative self-focused attention and fear of evaluation, thereby being linked to the maintenance of social anxiety in vulnerable populations (Thorisdottir et al., 2020).

Taken together, these findings suggest that the differences observed between clusters reflect not only varying levels of SM use, but also qualitatively distinct patterns of interpersonal difficulties and social anxiety, which may be associated, in part by gender. This underscores the importance of viewing SM use profiles as complex configurations that integrate behavioural and interpersonal characteristics, rather than focusing solely on the amount of time spent on digital platforms.

At the same time, these findings suggested that although intensive SM use may be associated with interpersonal difficulties, it does not in itself account for them. Rather, additional factors should be considered, including what adolescents do online, their motivations for using particular platforms, and what they derive from these activities. In this regard, previous research has highlighted the possibility of reverse or bidirectional associations, whereby psychological difficulties may also influence patterns of social media use (Hartanto et al., 2021).

Understanding these differences requires attention to multiple aspects. Recent research on SM use has shown that females tend to spend more time on SM and engage more intensively with its features (Boursier et al., 2020; Hassan & Afzal, 2022), whereas males tend to devote more time to gaming and instrumental or recreational online activities (Twenge et al., 2020).

Moreover, the number of accounts a user maintains across one or more SM platforms may partly explain the difficulties they experience online (Boer et al., 2021; Coyne et al., 2020; Primack et al., 2017). The use of alternative profiles and secondary accounts is becoming increasingly common but remains under-researched (van der Nagel, 2018).

Despite the contributions of the present study to understanding patterns of social media use in adolescence, several limitations should be acknowledged. First, data were collected using self-report measures, which may be affected by biases such as social desirability and limitations in participants’ self-perception. Second, the cross-sectional design precludes any inference about causality or directionality, and the observed associations may reflect bidirectional or alternative relationships. Third, although the cluster analysis allowed for the identification of distinct profiles, the overall cluster quality was moderate; therefore, the solution should be interpreted with caution and understood as representing probabilistic groupings rather than clearly defined categories, in line with the exploratory nature of person-centred approaches. Finally, although several statistically significant differences between clusters were identified, the associated effect sizes were generally small, indicating modest differences between groups and suggesting that the findings should be interpreted as general tendencies rather than strong or clearly differentiated patterns.

Further research should aim to replicate these findings and assess the stability of the cluster solution in independent samples. It would also be important to examine whether similar profiles emerge when sex is not included as a clustering variable. Incorporating multi-method approaches, including behavioural or informant-based measures, would strengthen the validity of the findings. Such efforts will contribute to refining and validating social media use profiles, given their relevance for promoting healthy patterns of use. This is particularly relevant in adolescent populations and for extreme-use groups, such as the outlier group identified in the present study. Additionally, increasing the sample size would allow for more detailed comparisons between groups and a better understanding of their psychological correlates.

## 5. Conclusions

In conclusion, the findings highlight the value of addressing adolescent SM use from a differentiated, profile-based perspective that considers not only the intensity of use, but also patterns of use based on the variables assessed in the present study. The identification of distinct usage profiles and their associations with interpersonal difficulties and social anxiety may contribute to informing the development of preventive and educational internet use programmes that promote healthy internet use. In line with recent evidence, the impact of SM on adolescent mental health depends less on the amount of time spent online and more on who is using it, how it is used, and for what purposes (Valkenburg et al., 2022). Accordingly, these findings should be interpreted within this broader framework, without assuming causal relationships. Moreover, given the person-centred and exploratory nature of the study, the identified profiles should be understood as provisional patterns that require further empirical validation. Acknowledging this complexity is essential for promoting more adaptive patterns of SM use and for supporting psychological well-being during a developmental period that is particularly sensitive to social influences.

The findings may have important implications for clinical, educational and preventive contexts. They showed that SM use profiles are differentially associated with levels of interpersonal difficulties and social anxiety, rather than with undifferentiated difficulties. However, given the cross-sectional nature of the study, these associations should not be interpreted as indicative of directionality or causality. Future research is needed to replicate these findings in independent samples and to examine the stability and generalisability of the identified profiles. Incorporating education on SM—its use, benefits and risks—in mental health promotion programmes may contributing to help support adolescent well-being.

## Figures and Tables

**Table 1:** Interpretation of IIP-32 scales (Barkham et al., 1996; Salazar et al., 2010).

Scale	Interpretation
IIP-1 Domineering/Controlling	Higher scores indicate difficulties related to control and a tendency to attempt to influence or change others’ behaviour. Individuals with high scores describe themselves as overly controlling or manipulative.
IIP-2 Vindictive/Self-centred	Higher scores reflect difficulties characterised by mistrust and suspicion towards others, together with a reduced capacity to consider others’ needs and well-being. They are also associated with a marked tendency towards anger and irritability, a propensity to hold grudges, difficulty forgiving, and preoccupation with revenge, which may contribute to frequent interpersonal conflict and limit social functioning.
IIP-3 Cold/Distant	Higher scores indicate a limited capacity to express or experience affection towards others, difficulties with commitment, and reduced willingness to be generous, get along with others, or forgive. Such individuals may also present as emotionally distant.
IIP-4 Socially Inhibited	Higher scores indicate social inhibition and heightened sensitivity to negative evaluation, with individuals experiencing anxiety, shyness, and embarrassment in the presence of others. Such scores are also associated with difficulties initiating social interactions, expressing feelings, integrating into groups, and functioning comfortably in social contexts.
IIP-5 Non-assertive	Higher scores reflect difficulties in expressing one’s own needs, discomfort in assuming positions of authority, and a marked lack of firmness and assertiveness, often associated with low self-esteem and limited self-confidence. Individuals with higher scores may appear insecure, avoid taking initiative or being the centre of attention, and tend to avoid situations involving social challenges or the exercise of influence over others.
IIP-6 Overly Accommodating	Higher scores describe individuals who find it difficult to experience or express anger due to fear of offending others. They tend to characterise themselves as trusting and easily misled, as well as overly accommodating, deferential, and excessively kind or submissive. They may also be reluctant to say “no” to others and are easily led.
IIP-7 Self-sacrificing	Higher scores indicate individuals who make excessive efforts to please others and who may be overly generous, trusting, affectionate, and permissive in their interactions. They may also display an excessively affiliative interpersonal style.
IIP-8 Intrusive/Needy	Higher scores describe individuals who are friendly, outgoing, and sociable, with a strong need for connection with others and difficulty being alone. Their desire for attention may lead them to share personal information excessively or inappropriately, and they may experience difficulties maintaining boundaries and preserving privacy.

**Table 2:** Comparison of clustering variable descriptive statistics across groups.

Clustering Variables	Group 1 *n* = 49 (16.2%)	Group 2 *n* = 111 (36.6%)	Group 3 *n* = 79 (26.1%)	Group 4 *n* = 51 (16.8%)	Outlier Group *n* = 13 (4.3%)
Sex	% (*n*)	% (*n*)	% (*n*)	% (*n*)	% (*n*)
- Female	100% (49)	100% (111)	0%	0	5.3% (9)
- Male	0%	0%	100% (79)	100% (51)	3% (4)
Time spent on SM	% (*n*)	% *(n*)	% (*n*)	% (*n*)	% (*n*)
- I only use the internet to access SM	8.3% (1)	0%	0%	41.7% (5)	50% (6)
- I spend over half my internet time on SM	0%	57.5% (111)	40.9% (79)	0%	1.6% (3)
- I spend the same amount of time SM as on other online sites	55.4% (36)	0%	0%	44.6% (29)	0%
- I spend less than half my time online on SM	36.4% (12)	0%	0%	51.5% (17)	12.1% (4)
Number of accounts on preferred SM	% (*n*)	% (*n*)	% (*n*)	% (*n*)	% (*n*)
- 1	65.3 (32)	55.9 (62)	72.2 (62)	68.6 (35)	
- 2	28.3 (14)	36 (40)	22.8 (18)	31.4 (16)	
- ≥3	6.1 (3)	8.1 (9)	5.1 (4)		
	*M* (*SD*)	*M* (*SD*)	*M* (*SD*)	*M* (*SD*)	*M* (*SD*)
Number of accounts on preferred SM	1.41 (0.61)	1.55 (0.72)	1.34 (0.62)	1.31 (0.47)	3.46 (1.85)
Hours spent daily on SM	2.27 (1.43)	3.77 (2.22)	3.39 (1.90)	2.27 (1.02)	5.38 (2.18)

**Table 3:** Comparison of IIP-32 across groups.

IIP-32	Group 1	Group 2	Group 3	Group 4	Test Statistic	*ω* ^2^
	*n* = 49 (16.2%)	*n* = 111 (36.6%)	*n* = 79 (26.1%)	*n* = 51 (16.8%)		
	*M* (*SD*) [CI 95%]	*M* (*SD*) [CI 95%]	*M* (*SD*) [CI 95%]	*M* (*SD*) [CI 95%]	F (*p*)	
IIP-1	1.90 (2.33) [1.23–2.57]	2.96 (2.83) [2.43–3.50]	2.43 (2.67) [1.83–3.03]	2.49 (2.29) [1.85–3.13]	2.07 (0.107)	0.010
IIP-2	2.69 (2.80) [1.89–3.50]	3.38 (3.80) [2.66–4.09]	3.97 (3.37) [3.22–4.73]	* 4.96 (4.30) [3.75–6.17]	3.80 (0.012)	0.027
IIP-3	4.73 (3.34) [3.77–5.70]	5.57 (3.86) [4.48–6.29]	4.46 (3.70) [3.63–5.29]	5.16 (3.93) [4.05–6.26]	1.48 (0.223)	0.005
IIP-4	7.20 (4.29) [5.97–8.44]	6.45 (3.72) [5.75–7.15]	6.00 (3.89) [5.13–6.87]	6.53 (4.38) [5.30–7.76]	0.85 (0.468)	0.00
IIP-5	6.27 (3.48) [5.26–7.27]	6.15 (3.56) [5.48–6.87]	5.66 (2.86) [5.02–6.30]	5.76 (3.97) [4.61–6.84]	0.57 (0.635)	0.00
IIP-6	* 7.80 (3.47) [6.80–8.79]	6.82 (3.71) [6.12–7.52]	6.29 (2.91) [5.64–6.94]	5.63 (2.79) [4.84–6.41]	4.30 (0.006)	0.030
IIP-7	8.45 (3.58) [7.42–9.48]	* 9.04 (3.70) [8.34–9.73]	7.20 (3.03) [6.52–7.88]	6.90 (3.44) [5.93–7.87]	6.60 (<0.001)	0.055
IIP-8	3.71 (2.88) [2.89–4.54]	* 5.15 (3.33) [4.53–5.78]	4.97 (3.03) [4.30–5.65]	3.94 (2.83) [3.14–4.74]	3.83 (0.011)	0.027
IIP-Total	42.76 (14.04) [38.72–46.79]	* 45.52 (15.72) [42.57–48.48]	40.99 (15.56) [37.5–44.47]	41.33 (13.23) [36.49–46.18]	1.53 (0.208)	0.006

Note: IIP-1: Domineering/Controlling (PA); IIP-2: Vindictive/Self-Centred (BC); IIP-3: Cold/Distant (DE); IIP-4: Socially Inhibited (FG); IIP-5: Non-assertive (HI); IIP-6: Overly Accommodating (JK); IIP-7: Self-Sacrificing (LM); IIP-8: Intrusive/Needy (NO). Post hoc tests: * IIP-2: G4 > G1 (*p* = 0.012); IIP-6: G1 > G4 (*p* = 0.005); IIP-7: G2 > G3 (*p* < 0.001), G2 > G4 (*p* = 0.003). IIP-8: G2 > G1 (*p* = 0.033).

**Table 4:** Comparison of SAQ-A30 across groups.

SAQ-A30	Group 1	Group 2	Group 3	Group 4	Test Statistic	*ω* ^2^
	*n* = 49 (16.2%)	*n* = 111 (36.6%)	*n* = 79 (26.1%)	*n* = 51 (16.8%)		
	*M* (*SD*) [CI 95%]	*M* (*SD*) [CI 95%]	*M* (*SD*) [CI 95%]	*M* (*SD*) [CI 95%]	F (*p*)	
Factor 1	19.14 (6.13) [17.38–20.90]	* 19.21 (6.07) [18.06–20.35]	16.25 (5.02) [15.13–17.38]	15.37 (5.95) [13.70–17.05]	7.79 (<0.001)	0.067
Factor 2	16.47 (6.68) [14.51–18.35]	15.52 (5.39) [14.41–16.44]	13.82 (5.22) [12.65–14.99]	14.25 (5.53) [12.68–15.79]	2.53 (0.600)	0.018
Factor 3	20.22 (5.77) [18.57–21.88]	* 20.41 (5.63) [19.35–21.46]	18.41 (5.17) [17.25–19.56]	17.63 (6.38) [15.83–19.42]	3.75 (0.013)	0.030
Factor 4	17.45 (5.34) [15.91–18.98]	* 17.46 (5.40) [16.44–18.48]	15.86 (4.16) [16.44–18.48]	15.31 (4.96) [13.92–16.71]	3.24 (0.024)	0.023
Factor 5	19.35 (4.7) [18.00–20.70]	* 20.02 (4.55) [19.16–20.87]	17.94 (4.20) [16.99–18.88]	17.63 (4.52) [16.36–18.90]	5.05 (0.002)	0.040

Note: F1: Public speaking/Interaction with figures of authority; Factor 2: Interaction with strangers; Factor 3: Interaction with people I find attractive; Factor 4: Assertive expression of displeasure or anger; Factor 5: Fear of embarrassment or appearing foolish. Post hoc tests * Factor 1 G1 > G3 (*p* = 0.034); G1 > G4 (*p* = 0.013; G2 > G3 (*p* = 0.002); G2 > G4 (*p* < 0.001); Factor 3 G2 > G4 (*p* = 0.044); Factor 5 G2 > G3 (*p* = 0.008) G2 > G4 (*p* = 0.013).

**Table 5:** Group characteristics based on the clustering variables and between-group differences in interpersonal difficulties and social anxiety.

	Group 1	Group 2	Group 3	Group 4	Outlier Group
	Moderate-integrated female users	Females with intensive SM-centred use	Males with intensive SM use	Males with peripheral SM use	Males and Females with extreme SM use
IIP-32 Scores	IIP-6 Overly Accommodating	IIP-7 and 8. Self-Sacrificing and Intrusive/Needy	Lowest score on interpersonal difficulties (IIP-Total)	IIP-2 Vindictive/Self-Centred	This group was not included in inferential analyses
SAQ-A30 Scores	Fear of social evaluation, public speaking, interactions with members of the opposite sex, and fear of being embarrassed or ridiculed		Between-group differences indicated lower scores across all factors		
Description	Integrated SM use, with an impact on social interaction and interpersonal difficulties, marked by overly accommodating behaviour	Largest group: intensive SM use and high interpersonal vulnerability with marked sensitivity to social evaluation (social anxiety).	Intensive SM use, with lower interpersonal difficulties and lower social anxiety	Heterogeneous group: interpersonal difficulties with Vindictive/Self-Centred profile and lower social anxiety	An extreme, non-representative profile, potentially at risk

## Data Availability

The raw data (de-identified data and analysis scripts) supporting the conclusions of this article will be made available by the authors on request.
